# Urological manifestations of Chikungunya fever: A single centre experience

**DOI:** 10.4103/0974-7796.68859

**Published:** 2010

**Authors:** Ramen Baishya, Vikas Jain, Arvind Ganpule, Veeramani Muthu, Ravindra B. Sabnis, Mahesh R. Desai

**Affiliations:** Department of Urology, Muljibhai Patel Urological Hospital, Nadiad - 387 001, Gujarat, India

**Keywords:** Chikungunya fever, lower urinary tract symptoms

## Abstract

**Background:**

Chikungunya is a viral infection often associated with lower urinary tract dysfunction. This study evaluates the urological squeal of Chikungunya fever in a single centre after an epidemic in 2006–2007 in India.

**Materials and Methods:**

Retrospective analysis of medical records of 13 patients with lower urinary tract symptoms after Chikungunya fever was evaluated and outcome following intervention assessed.

**Results:**

A total of 13 patients (M:F=9:4), with age ranging from 30 to 72 years, were included in the study. They presented with chronic urinary retention (n=9, 69.23%) of which two had paraparesis, voiding symptoms alone (n=7, 53.8%), storage symptoms alone (n=3, 23%), and acute urinary retention (n=1, 7.6%). Presentation with lower urinary tract symptoms after an episode of Chikungunya fever was after a mean period of 163 days (range 30-360 days). Mean serum creatinine on presentation was 1.8 mg/dl (0.6–6.5 mg/dl). Evaluation revealed dilated upper tract in four (30.7%) patients. Cystometrography showed acontractile detrusor (n=3, 37.5%), hypocontractile detrusor (n=3, 37.5%), overactive detrusor (n=1, 12.5%) and normal study (n=1, 12.5%). At the mean follow up of 11 months, 11 patients (84.6%) had satisfactory functional outcome after intervention, namely supra pubic diversion and bladder training (n=5, 38.4%), alpha blocker (n=3, 23%), timed frequent voiding (n=2, 15.3%), clean intermittent catheterization (n=2, 15.3%), trial void with alpha blocker (n=1, 7.6%) while two are on continuing supra pubic diversion due to persistent neurological deficit.

**Conclusions:**

Chikungunya fever is an uncommon entity in urological practice, often associated with urinary symptoms. An accurate assessment of the symptoms and timely intervention prevents upper tract deterioration and improves the quality of life.

## INTRODUCTION

Chikungunya is an acute viral infection of abrupt onset, heralded by fever and severe arthralgia, followed by other constitutional symptoms and rash. It lasts for about a period of 1–7 days. This acute phase lasts for 2–3 days. The temperature may remit for 1 or 2 days, resulting in “saddle back” fever curve. Though case diagnosis can only be made by laboratory means, Chikungunya should be suspected when epidemic occurs with the characteristic triad of fever, rash and rheumatic manifestations.[1]

Chikungunya virus (CHIKV) is a Group IV (+) (RNA) belonging to family Togaviridae with genus *Alphavirus* and species CHIKV. Chikungunya virus is most commonly transmitted to humans through the bite of an infected mosquito, specifically mosquitoes of the *Aedes* genus, which usually bite during daylight hours.[2] Neuro-invasive cases and hemorrhagic manifestation related to CHIKV infection have been conclusively documented in scientific literature.[3] In India, epidemics of Chikungunya fever were reported during the last millennium, viz., 1963 (Kolkata), 1965 (Pondicherry and Chennai in Tamil Nadu; Rajahmundry, Visakhapatnam and Kakinada in Andhra Pradesh; Sagar in Madhya Pradesh and Nagpur in Maharashtra) and 1973 (Barsi in Maharashtra). Thereafter, sporadic cases continued to be recorded especially in Maharashtra state during 1983 and 2000 (in Yawat).

The virus can cause encephalitis, myelopathy and neuropathy and combinations thereof. Involvement of bladder is thought to be due to myelopathy which occurs in about 44.6% of patients. In this study, we found that it started a few days after fever. Patient often first developed retention of urine and then showed paraparesis. Usually, the upper limbs were more or less not involved.[4–6]

This study evaluates the urological sequelae of Chikungunya fever in a single centre after an epidemic in 2006–2007 in India.

## MATERIALS AND METHODS

After the epidemic of Chikungunya fever in 2006–2007, we noticed a subset of patients who developed lower urinary tract symptoms after the fever. Although there were lots of suspected cases of Chikungunya fever with lower urinary tract symptoms, only serologically confirmed cases without prior history of lower urinary tract symptoms were included in the study. At the initial presentation, the patients were evaluated in outpatient department with detailed history, International Prostatic Symptom Score (IPSS), physical examination including digital rectal examination and focused neurological examination. Then, urine bacteriologic studies, renal function test, ultrasonography, and uroflowmetry were performed. Seven patients were subjected to cystometrographic study. The patients were followed regularly with clinical reassessment, IPSS score, renal function test, urine bacteriologic studies and ultrasound.

Retrospective analysis of their clinical history and physical examination was critically analyzed in terms of demographics, laboratory parameters, treatment offered and outcomes.

## RESULTS

### Demographics

**Figure 1:**
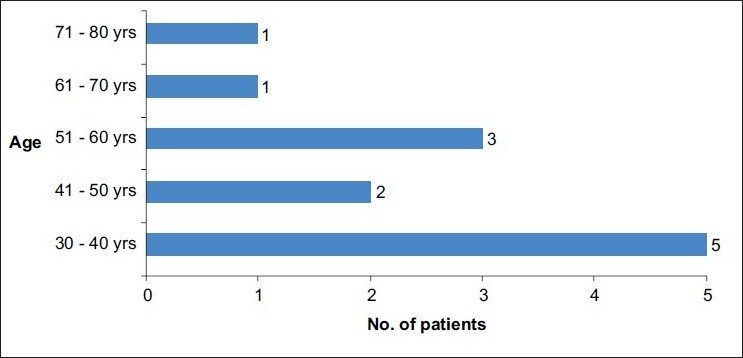
Decade wise presentations of patients

There were 13 patients in the study group. Age ranged from 30 to 70 years with a mean age of 39.2 years. There were nine males and four females with lower urinary tract symptoms (LUTS). Decade wise distribution of patients is given in Figure 1.

### Lower urinary tract symptoms

Mean days of presentation after the febrile episode was 163 days ranging from 30 to 360 days. Seven patients (53.8%) presented with chronic retention of urine. Only one patient presented with acute retention of urine. The mean IPSS (questions 1–7) of the patients was 21 (range 13–34). Three patients (23%) had only storage symptoms (IPSS subscore, questions 2, 4, 7), seven patients (53.8%) had voiding symptoms only (IPSS subscore questions 1, 3, 5, 6).

### Laboratory and ultrasonography findings

Mean serum creatinine of the patients at presentation was 1.8 mg/dl (0.6–6.5 mg/dl). Four patients had raised serum creatinine, i.e., above 1.5 mg/dl (mean 2.8 mg/dl). Total four patients (30.76%) had bilateral hydroureteronephrosis. Mean prostate size on abdominal sonography was 29.7 g (10–35 g) in nine male patients. Five patients (38.4%) had significant growth in urine culture (more than 10^5^ colony forming units per ml). The organism isolated was *Escherichia coli* (100%).

### Uroflowmetry

Uroflowmetry was available in 12 patients. Most of them had poor flow with high residue urine. The parameters are shown in Table 1.

**Table 1:** Parameters of uroflowmetry in 12 patients

	Male (n=8)	Female (n=4)
Mean Q max (ml/second)	13 (5.3–32)	15.2 (7–38.2)
Mean voided volume (ml)	182 (40–826)	212 (160–260)
Mean post void residue (ml)	84 (10–160)	68 (8–100)

### Cystometrography

Experienced investigators performed this study in seven patients, who were in line with the suggested good urodynamic practice standards of the International Continence Society. One patient approached us with urodynamic study done elsewhere. Cystometrographic examinations were performed with external pressure transducers, 6 and 8 Fr. transurethral and 10 Fr. single lumen rectal catheters. Sterile physiological saline at a temperature of 37°C was infused through the transurethral catheter at a filling rate of 10% of expected capacity per minute. The cystometrographic traces were judged by the investigator together with a senior urologist according to the 2002 classification of the International Continence Society. The results of this study are presented in Table 2.

**Table 2:** Cystometrographic findings

Underactive detrusor (%)	3 (37.5)
Acontractile detrusor (%)	3 (37.5)
Overactive detrusor (%)	1 (12.5)
Normal study (%)	1 (12.5)

### Management

The lone male patient with acute retention was catheterized. Alpha blocker (Tamsulosin 0.4 mg) was started and trial void was given after 1 week. He voided successfully but was keeping significant post void residue (>10% of voided volume). Alpha blocker was continued for 3 months, subsequent to which he was voiding to completion on follow up.

In five male patients with chronic retention, supra pubic catheter (SPC) was placed. Bladder training was taught after 6 weeks. Although initially the residue was significant, gradually it decreased. SPC was removed once the postvoiding residue (PVR) was below 50 ml after a mean duration of 3 months. However, two patients are still on regular monthly SPC change due to persistent voiding symptoms without significant improvement. Two female patients with chronic retention were taught to empty their bladder by doing clean intermittent catheterization (CIC) after voiding per urethra. They were asked to stop doing CIC once PVR was less than 50 ml. Mean duration of CIC was 3.5 months. Two patients were symptom free after starting alpha blocker (Tamsulosin 0.4 mg). Mean duration of treatment was 5 months. Two patients with minimal symptoms (mean IPSS: 11) were asked to do time frequent double voiding. The patient with overactive bladder was symptom free after 3 months of Oxybutynin hydrochloride (Dose: 0.1 mg/kg/day).None of the patients had any deterioration in the renal function in the follow up. The four patients whose initial serum creatinine was high (mean 2.8 mg/dl) did not require any renal replacement therapy in the follow up, maintaining a mean serum creatinine of 2.1 mg/dl till the last follow up.

**Figure 2:**
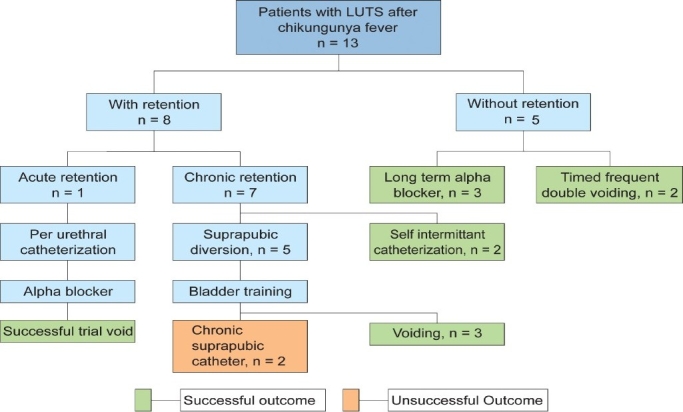
Management and results

We followed up all the patients for a mean of 11 months (1–24 months). Eleven patients (84.6%) had successful outcome defined by voiding to completion without any assistance while maintaining stable renal function. Our approach to the patients and results are detailed in Figure 2.

## DISCUSSION

**Figure 3:**
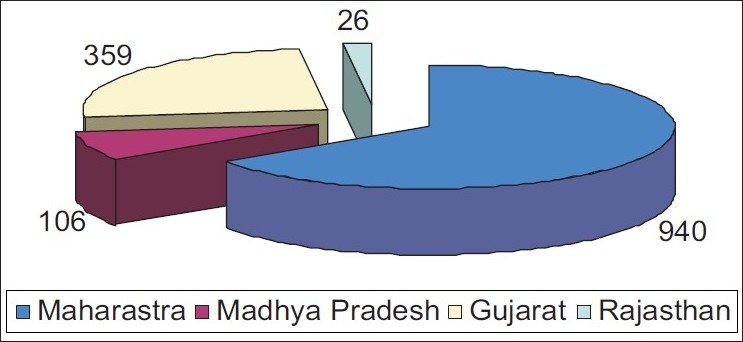
Number of confirmed cases in Gujarat and three nearby states during 2006–2008

After quiescence of three decades, in 2006, Chikungunya outbreak was reported in India.[1] A total number of 3871 confirmed cases of Chikungunya had been reported from 17 states in India during 2006–2008.[3] Gujarat state alone had 9.27% of all confirmed cases. Figure 3 shows the number of confirmed cases in Gujarat and three nearby states. A lot of patients presented to urology outpatient department with lower urinary tract symptoms. However there is paucity of literature regarding urological manifestations of Chikungunya fever. In our belief, it is the first study of its kind, exploring the connection between Chikungunya fever and lower urinary tract symptoms. The urinary symptoms which are thought to be due to myelopathy were found in less than 50% of the patients.[4] In our study, the most common presentation was chronic urinary retention. We observed that the cause of chronic retention is acontractility or underactivity of detrusor, as evident on urodynamic study. Cystometrography appears to be very helpful to assess the degree of bladder involvement in patients with chronic retention and also to detect improvement on subsequent follow up. Female patients with underactive detrusor can be managed solely with self-intermittent catheterization with durable response. Patients with acontractile detrusor usually require prolonged bladder training to achieve unassisted voiding. In these groups of patients SPC was found to be very helpful, as initially it decompresses the system and latter on it can be used to evacuate the post void residue. Objective evidence of recovery of bladder can be achieved by reduction in post void residual volume. Tamsulosin was found to be effective in patients with moderate symptoms and patients with acute urinary retention. Most of the patients who were treated by alpha blocker alone or clean intermittent self-catheterization recovered well. The two patients who are still on supra pubic diversion are old (mean age 76.6 years) and had paraparesis precluding the possibility of CIC. One of them have hypointense lesion in the spinal cord in magnetic resonance imaging (MRI) study, suggestive of myelopathy possibly as a sequelae of Chikungunya fever.[5] The contractility power of bladder recovered spontaneously over a period of time in majority of patients as evident from the fact that they could (*n*=11, 84.6%) achieve near completion voiding during follow up. We believe that they require bladder evacuation therapy till the time of disappearance of neurological deficit to protect upper tract and permanent nephron damage as renal function of these four patients remained stable and did not require any further intervention.

The major limitations of the current study were small numbers of patient group, retrospective nature of the study, short period of follow up and absence of electromyography evaluation.

All the patients were initially treated by primary care physician and approached for urological consultation due to persistence of urinary symptoms. Hence, it is difficult to comment on the initial severity of the urinary symptoms associated with Chikungunya fever. A prospective study might throw light on the genesis of urinary involvement among patients with Chikungunya fever and its progression, in due course of time.

## CONCLUSIONS

Chikungunya fever is an uncommon entity in urological practice, often associated with urinary symptoms. An accurate assessment of the symptoms and timely intervention prevent upper tract deterioration and improve the quality of life.
